# Mutation of RNA Pol III Subunit *rpc2/polr3b* Leads to Deficiency of Subunit Rpc11 and Disrupts Zebrafish Digestive Development 

**DOI:** 10.1371/journal.pbio.0050312

**Published:** 2007-11-27

**Authors:** Nelson S Yee, Weilong Gong, Ying Huang, Kristin Lorent, Amy C Dolan, Richard J Maraia, Michael Pack

**Affiliations:** 1 Department of Medicine, University of Pennsylvania School of Medicine, Philadelphia, Pennsylvania, United States of America; 2 National Institute of Child Health and Human Development, National Institutes of Health, Bethesda, Maryland, United States of America; 3 Department of Cell and Developmental Biology, University of Pennsylvania School of Medicine, Philadelphia, Pennsylvania, United States of America; Wellcome Trust Sanger Institute, United Kingdom

## Abstract

The role of RNA polymerase III (Pol III) in developing vertebrates has not been examined. Here, we identify a causative mutation of the second largest Pol III subunit, *polr3b*, that disrupts digestive organ development in zebrafish *slim jim* (*slj*) mutants. The *slj* mutation is a splice-site substitution that causes deletion of a conserved tract of 41 amino acids in the Polr3b protein. Structural considerations predict that the *slj* Pol3rb deletion might impair its interaction with Polr3k, the ortholog of an essential yeast Pol III subunit, Rpc11, which promotes RNA cleavage and Pol III recycling. We engineered Schizosaccharomyces pombe to carry an Rpc2 deletion comparable to the *slj* mutation and found that the Pol III recovered from this *rpc2*-Δ yeast had markedly reduced levels of Rpc11p. Remarkably, overexpression of cDNA encoding the zebrafish *rpc11* ortholog, *polr3k*, rescued the exocrine defects in *slj* mutants, indicating that the slj phenotype is due to deficiency of Rpc11. These data show that functional interactions between Pol III subunits have been conserved during eukaryotic evolution and support the utility of zebrafish as a model vertebrate for analysis of Pol III function.

## Author Summary

The transmission of genetic information from DNA to messenger RNA to protein depends on the function of a large number of small noncoding RNA molecules. The genes encoding these RNAs are transcribed by RNA polymerase III (Pol III), a 17-subunit protein complex whose structure is closely related to that of RNA polymerases I and II. Here, we report the effect of a mutation in a gene encoding one Pol III subunit, Polr3b, which disrupts proliferation and growth of tissue progenitor cells in the zebrafish digestive system. Analyses of a nearly identical mutation in the yeast S. pombe gene encoding Polr3b, also known as Rpc2, suggested that the zebrafish mutation disrupted the mutant Polr3b protein's interaction with another Pol III subunit, Polr3k, also known as Rpc11. Overexpression of the gene encoding Polr3k in the Polr3b mutants partially rescued (reversed) the mutant phenotype. These findings extend our knowledge of the mechanism of Pol III function, which appears to have been highly conserved during eukaryotic evolution. Furthermore, these data also suggest that assembly of the 17-subunit Pol III enzyme is a dynamic process, since Polr3k overexpression can partially rescue the mutant phenotype. Understanding how Pol III is assembled has implications for human disease, since Pol III activity is markedly increased in most cancers.

## Abstract 3

Transcription of tRNAs and other noncoding RNAs by RNA polymerase III is essential for cell proliferation, growth, and survival. With the aide of experiments in the fission yeast,*S. pombe*, the authors report how a small in-frame deletion in the second largest Pol III subunit, Polr3b, affects tissue progenitor cells in the developing zebrafish digestive system.

## Introduction

RNA Polymerase III (Pol III) is a 17-subunit complex that is responsible for the transcription of small noncoding RNAs such as transfer RNAs (tRNAs), 5S ribosomal RNA (rRNA), U6 small nuclear RNA (snRNA), 7SL RNA, and others in eukaryotes [1,2]. The two largest subunits, Rpc1 (∼160 kDa) and Rpc2 (∼130 kDa), are highly homologous to their counterparts in Pol I and Pol II, and together provide a large surface area for interaction with many of the other subunits [2]. Structural analyses of Pol III complexes [3,4], together with two-hybrid analysis [5], have identified multiple subunit interactions (reviewed in [1]). These, together with biochemical and genetic analyses, have led to a model that attributes some of the unique functions of Pol III, including its high processivity, efficient transcription termination and recycling activity, RNA 3′ cleavage activity, and interaction with diverse promoters, to specific individual subunits.

Mutational analyses in yeast clearly show that an intact Pol III system is essential for cell growth. The effects of reduced Pol III function are predicted to be broad, including protein synthesis necessary for cell-cycle progression (tRNAs), ribosome biogenesis (5S rRNA), mRNA splicing (U6 snRNA), and membrane targeting of newly translated proteins (7SL RNA). Pol III transcription is tightly regulated during the cell cycle [6] and in response to cellular stress [7]. Recent studies in human cells have also highlighted the roles of oncogenes and tumor suppressors such as Rb [8,9], p53 [9–11], and cMyc [9,12] in controlling the interactions between the transcription factors that bring the Pol III complex to the promoters of its target genes (reviewed in [13,14]). Other proteins, such as Maf1 [15–18] and the oncogenic kinase CK2 [19–20], can regulate Pol III function through direct interactions with the Pol III complex. Thus, eukaryotic cells have evolved multiple independent mechanisms for regulating Pol III activity.

Given the importance of Pol III for cell growth and proliferation, it is not surprising that it is deregulated in cancers and in cells transformed by viral oncoproteins [13,14]. These findings suggest that it may be possible to disrupt transformed cells by inhibiting Pol III function. It is not known, however, whether Pol III inhibition has deleterious effects in nonproliferating cells of complex multicellular organisms. Here, we describe the positional cloning of a mutation, *slim jim* (*slj^m74^*; hereafter, *slj*; [21–23]), that targets *polr3b*, the zebrafish ortholog of a yeast Pol III subunit gene *rpc2*, which is highly conserved in eukaryotes. tRNAs and other Pol III transcripts are decreased in *slj* larvae. Accordingly, the *slj* mutation has a pronounced effect on the growth and proliferation of progenitor cells in the digestive tract of *slj* larvae, but surprisingly, no overt effect on the survival of cells in other nonproliferating mutant tissues at the same developmental stage. Also unexpectedly, the *slj* mutation does not interfere with differentiation of most epithelial lineages in the developing zebrafish intestine, but has a profound effect on epithelial cell morphology [22]. These data surprisingly indicate that specific cell types in the developing fish are differentially sensitive to the *slj* mutation and suggest that this may be due to different requirements for Pol III activity.

Structural comparisons of yeast Pol II and Pol III suggest that the *slj* mutation might perturb interaction of Rpc2 with Rpc11, an integral Pol III subunit that exhibits RNA 3′ cleavage activity and is required for efficient transcription recycling by Pol III [24–26]. Supporting this idea, we show that the Rpc11p protein is not present in the Pol III complex purified from S. pombe engineered to carry an Rpc2 deletion mimicking the *polr3b^slj^* allele. Microinjection of an overexpression construct encoding the zebrafish Rpc11 ortholog, Polr3k, suppresses the exocrine defects in *slj* larvae, indicating that the slj phenotype is due to deficiency of the Polr3b–Polr3k (Rpc2–Rpc11) interaction, and supporting broad conservation of Pol III structure in eukaryotes.

## Results

### Cell Type–Specific Reduction of Cell Proliferation in the Zebrafish Digestive Organs

**Figure 1:**
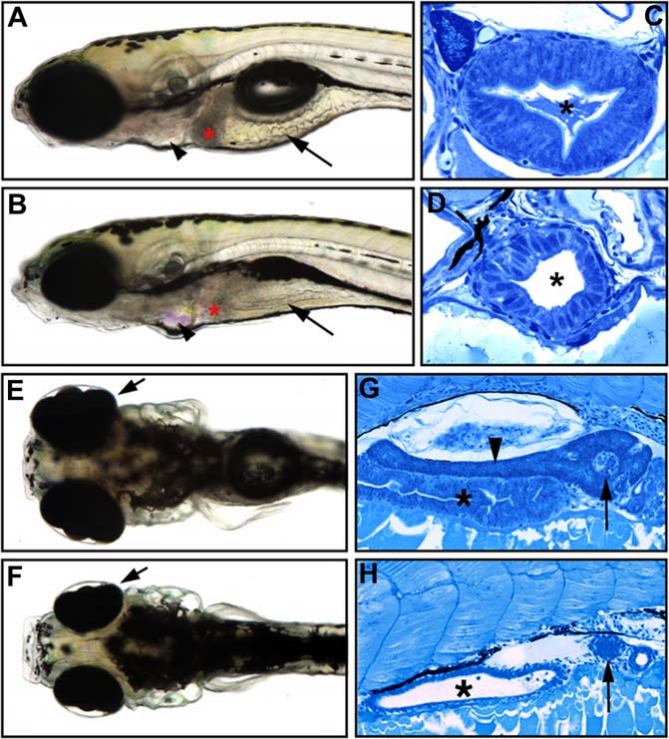
The *slj* Mutation Disrupts Intestinal and Exocrine Pancreas Development (A–D) Lateral views of 5-dpf wild-type (A) and *slj* (B) larvae, with representative histological cross-sections (C and D). The *slj* intestine is small, thin walled, and lacks folds (arrows, [A and B]) compared with wild type, and the *slj* terminal branchial arches are reduced (arrowheads). The columnar morphology and apical microvilli of the *slj* epithelium are less developed than in wild-type larvae (C and D); an asterisk (*) indicates the intestinal lumen. (E and F) The size of the *slj* liver (indicated by red asterisk in [A] and [B]) and the retinae (arrows, [E and F], dorsal view) are also reduced. (G and H) Although exocrine tissue is well developed in wild-type embryos (arrowhead in [G]), it is markedly reduced or absent in *slj* embryos (H); asterisks indicate intestine. By contrast, the *slj* and wild-type pancreatic islets are of comparable size (arrows).

The zebrafish *slj* mutation was recovered in a large-scale mutagenesis screen on the basis of altered intestinal morphology [21]. Compared to wild-type five-day post-fertilization (dpf) larvae, the intestine of 5-dpf *slj* larvae is small, thin walled, and lacks folds (Figure 1A and 1B). In histological sections, the *slj* intestinal epithelium appears immature compared with wild-type siblings (Figure 1C and 1D). Development of the exocrine pancreas is also severely affected by the *slj* mutation [23]. Little, if any, exocrine tissue is visible in histological sections of 5-dpf *slj* larvae (Figure 1G and 1H). By contrast, the pancreatic islet, which undergoes little or no expansion beyond the first 48 hours post-fertilization (hpf) appears normal in *slj* (Figure 1G and 1H).

To investigate the underlying cause of this feature of the slj phenotype, we measured *slj* intestinal and pancreas cell proliferation using bromodeoxyuridine (BrdU) and phospho-Histone H3 (PH3) immunohistochemistry. The BrdU assay revealed a nearly 2-fold reduction in the proportion of S-phase cells within the *slj* intestinal epithelium (InE; Table 1) and a nearly 5-fold reduction of the proportion of S-phase cells in the developing exocrine pancreas (ExP; Table 1) around the stage when the slj phenotype is first recognizable (72 hpf). By contrast, cell proliferation within the intestinal stroma was not affected by the *slj* mutation at this stage. Reduced proportion of intestinal epithelial and pancreatic S-phase cells, coupled with the normal percentage of M-phase cells at this stage (as determined by anti-PH3 immunohistochemistry), suggest the *slj* mutation causes a delay in the G1–S transition within highly proliferative organ progenitor cells. Consistent with this idea, the size of other highly proliferative tissues, such as the liver, retina, and terminal branchial arches, was also reduced in *slj* larvae (Figure 1E and 1F, and unpublished data).

**Table 1:**
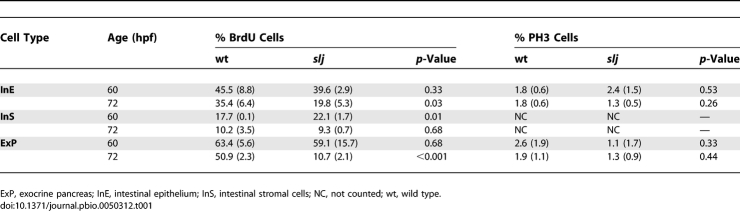
Cell Proliferation Indicies in Wild-Type and *slj* Larvae

### Causative Mutation of Zebrafish *polr3b* in Zebrafish *slj* Mutants

**Figure 2:**
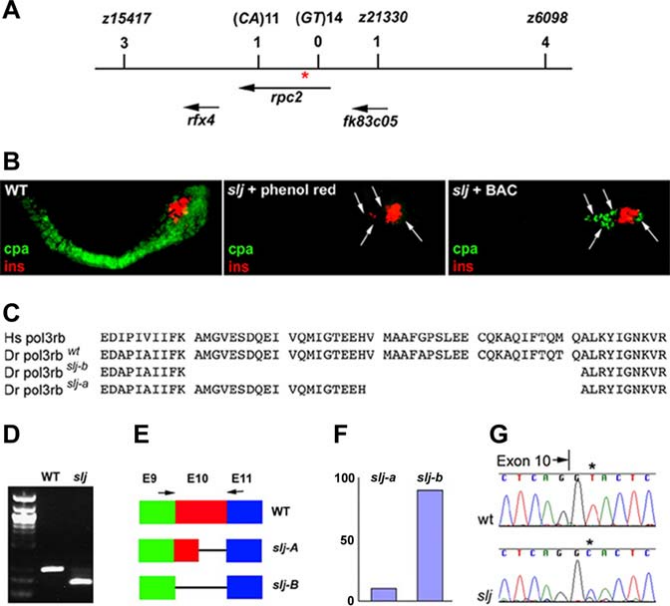
The *slj* Locus Encodes Zebrafish *polr3b*, the Gene Encoding the Second Largest Subunit of RNA Pol III (A) Schematic of the region surrounding the *slj* locus. Genetic markers and the number of recombinants are listed above the names of the genes and expressed sequence tags (ESTs) that map to this region of zebrafish Chromosome 18. A zero recombinant marker (*GT*)14, is located within the *polr3b* coding region. Arrows denote orientation of gene transcription. Red asterisk (*) denotes location of *slj* mutation. (B) High-power, lateral views of the immunostained pancreas from a 5-dpf wild-type zebrafish larva (left panel), and two *slj* larvae microinjected with either phenol red vehicle (middle) or the BAC spanning the *slj* locus (right panel). All larvae were processed for insulin (ins, red) and carboxypeptidase A (cpa, green) immunohistochemistry. A normal pattern of immunoreactive insulin is present in all larvae. BAC injection restores cpa staining and therefore partially rescues the *slj* exocrine (carboxypeptidase A) defect. Arrows point to cpa-positive exocrine pancreas cells in the BAC-injected larva and note their absence in the *slj* larva. (C) Predicted amino acid sequence encoded by the human (Hs) and zebrafish (Dr) Polr3b cDNA surrounding the region deleted by the *slj* mutation. Two *polr3b* mRNA splice variants are generated by the *slj* mutation: the *slj-b* variant lacks all 41 amino acids encoded by exon 10 (aa239–279) of the *polr3b* gene, whereas the *slj-a* variant lacks 22 exon 10 amino acids (aa258–279). (D) PCR amplification of cDNA derived from pooled 5-dpf *slj* larvae and homozygous wild-type (WT) siblings using primers that span *polr3b* exon 10, as depicted in (E). Sequencing revealed that a 278-bp band is amplified from wild-type larvae, whereas a 155-bp band corresponding to deletion of exon 10 is amplified from *slj* larvae. (E and F) Schematic (E) depicting the wild-type and *slj polr3b* mRNAs exons 9–11 (E9, E10, and E11). Arrows refer to PCR primers used to quantify the relative proportion of the slj-A and slj-B transcripts (F) in the 5-dpf *slj* and wild-type larvae shown in (D). (G) Schematic depiction of the DNA sequences encoded by wild-type and *slj polr3b* alleles. The *slj* mutation encodes a thymine (T) to cytosine (C) transition in the intron 10 splice acceptor.

To gain a better understanding of the cause of the proliferative defects associated with the slj phenotype, a positional cloning strategy was used to identify the targeted gene. Using bulk segregant analyses, we first identified a marker on Chromosome 18 linked to the *slj* locus (M. Mohideen, M. Fishman, and M. Pack; unpublished data). Mapping of subsequent markers identified a critical region surrounding the *slj* locus as described in Materials and Methods and Figure 2A. Ultimately, a bacterial artificial chromosome (BAC, zk103i16) spanning two flanking simple-sequence repeat markers (z15417 and z6098) was identified. Microinjection of the BAC DNA partially rescued *slj* exocrine pancreas defects (Figure 2B; *n* = 6 of 17 injected *slj* larvae), thus confirming that the BAC spanned the *slj* locus.

Sequence analysis from the zebrafish genome project identified three genes within BAC zk103i16 adjacent to the *slj* locus (Figure 2A). Further meiotic mapping narrowed the critical interval to a region that contained the *polr3b* gene (http://www.sanger.ac.uk/Projects/D_rerio). We then scanned *pol3rb* cDNA for mutations. Reverse-transcriptase PCR (RT-PCR) products and their sequencing identified a 123-bp deletion in *polr3b* cDNA amplified from *slj* mutant larvae, but not from homozygous wild-type larvae (Figure 2C and 2D). Additional analyses indicated that the 123-bp deletion corresponded precisely to exon 10 of the *polr3b* gene [27]. A smaller deletion, comprising 66 nucleotides from exon 10, was subsequently identified in a minority (∼10%) of the *polr3b* cDNA fragments (Figure 2E and 2F). Both deletions are predicted to occur in-frame, thus generating *polr3b* cDNAs encoding proteins truncated by internal deletion of either 22 or 41 amino acids.

Exon 10 skipping induced by the *slj* mutation could arise from disruption of the *polr3b* intron 10 splice acceptor. Sequence analyses confirmed this prediction: a thymine-to-cytosine transition was present in the intron 10 splice acceptor of the *polr3b^slj^*, but not the wild-type *polr3b* allele (Figure 2G). This single intronic substitution causes the exon 11 splice donor to utilize either the intron 9 splice acceptor, or a cryptic splice acceptor within exon 10, thus deleting either 123 or 66 exon 10 nucleotides from the mature *polr3b* mRNA (Figure 2C and 2E). Since the RT-PCR and sequencing analyses shown in Figure 2E and 2F were performed on whole embryos, the data indicate that the *slj* mutation leads to altered splicing with in frame codon deletions in the vast majority, if not all, of the *polr3b^slj^* transcripts.

**Figure 3:**
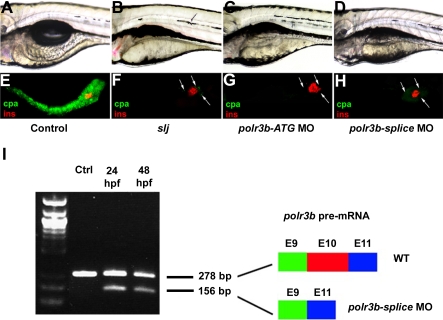
Antisense Knockdown of Zebrafish *polr3b* Generates a slj Phenocopy (A–D) Lateral views of 5-dpf and wild-type zebrafish larvae injected with vehicle (control [A]) or Morpholinos targeting either the *polr3b* translation initiation codon (*polr3b-ATG* MO [C]) or the *polr3b* exon 10 splice donor (*polr3b-splice* MO [D]). The intestine in both Morpholino-injected larvae resembles the *slj* intestine (B). (E–H) Corresponding images of the immunostained pancreas from these larvae: note that there is minimal exocrine tissue in larvae injected with either *polr3b* Morpholino, as occurs in *slj*. cpa, carboxypeptidase A; ins, insulin. (I) Agarose gel electrophoresis showing RT-PCR products amplified from *polr3b* cDNA derived from wild-type (WT) embryos injected with the *polr3b-splice* Morpholino. In both 24-hpf and 48-hpf larvae injected at the one-cell stage, a 155-bp band corresponding to in-frame deletion of exon 10 is evident (confirmed by DNA sequencing), but not in control larvae. The 278-bp wild-type fragment is also evident in the Morpholino-injected larvae.

To confirm that the *polr3b* mutation was responsible for the slj phenotype we designed antisense Morpholinos that targeted the *polr3b* mRNA (Figure 3). Microinjection of a Morpholino targeting the translation initiation codon (5′ ATG) led to early lethality (prior to 24 hpf) in the majority of injected embryos, with the remainder showing severe developmental delays (unpublished data). Injection of a lower dose of this Morpholino produced an slj intestinal and pancreatic phenocopy in approximately 50% and 60% of surviving wild-type 5-dpf larvae (*n* = 77 and 47 embryos analyzed, respectively, in two independent experiments; Figure 3A–3C and 3E–3G). We also designed a Morpholino spanning the intron 9 splice donor with the hope that targeting this site would induce deletion of exon 10. Indeed, RT-PCR and DNA sequence analysis of the *polr3b* cDNA derived from embryos microinjected with this Morpholino, but not control-injected embryos, revealed in-frame deletion of exon 10 of the *polr3b* cDNA (Figure 3I). Importantly, 52% and 43% of surviving embryos injected in two independent experiments showed an slj intestinal and pancreatic phenocopy (*n* = 33 and 53 total embryos analyzed, respectively; Figure 3D and 3H). In summary, mutant phenocopy by two nonoverlapping Morpholinos confirm identification of *polr3b* as the gene targeted by the *slj* mutation.

### *polr3b* Expression during Zebrafish Development

**Figure 4:**
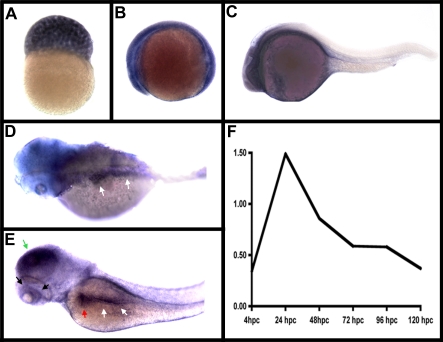
*polr3b* Expression in Developing Zebrafish (A) Strong maternal *polr3b* expression is evident at 3 hpf. (B) Widespread *polr3b* expression is evident at 12 hpf. (C) At 24 hpf, *polr3b* expression is most pronounced in the nervous system. (D) At 48 hpf, liver and intestinal *polr3b* expression (white arrows) is evident. There is also diffuse expression in the nervous system at this stage. (E) A similar pattern of *polr3b* expression is evident at 72 hpf (intestine and liver—white and red arrows, respectively). Retinal and nervous expression is also apparent at this stages (black and green arrows, respectively). Nervous system expression was variable at this stage, whereas digestive organ and retinal expression was consistent. (F) Quantitative RT-PCR data showing the relative expression levels of *polr3b* during embryonic and larval development. Expression levels of *polr3b* are relative to *hprt* expression.

To define the location and levels of *polr3b* expression in developing zebrafish embryos and larvae, we performed RNA whole-mount in situ hybridization assays and quantitative real-time RT-PCR amplification of the *polr3b* cDNA (Figure 4). These data showed strong maternal *polr3b* expression (Figure 4A) and strong zygotic *polr3b* expression that peaked at 24 hpf and subsequently declined (Figure 4B–4F). Beyond 3 dpf, we observed only low levels of *polr3b* expression in the digestive organs, that were only slightly above background and thus difficult to image (unpublished data). This decline in *polr3b* expression coincides with 5-fold reduced cell proliferation in the digestive system and other tissues between 2 dpf and 4 dpf [22]. This relatively low level of Polr3b expression may sensitize cells to the *slj* mutation, and provide an explanation as to why the digestive system and other proliferative tissue may be selectively affected in *slj* mutants (see Discussion).

**Figure 5:**
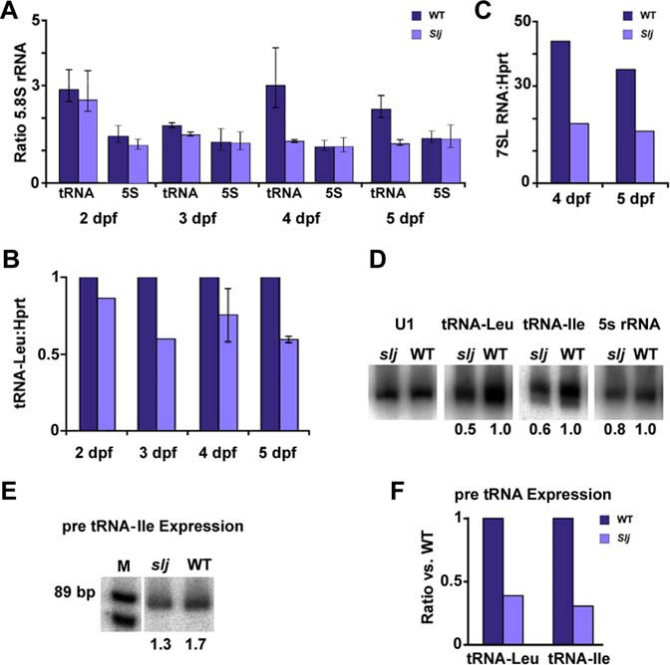
Reduced Pol III Target Gene Expression in *slj* Larvae (A) Total tRNA levels are reduced relative to the amount of 5.8S rRNA (a gene transcribed by Pol I) in pooled 4-dpf and 5-dpf *slj* larvae compared with wild-type (WT) siblings, whereas levels of 5S rRNA, another Pol III target gene product, are normal. Identical RNA samples analyzed in (A and B). tRNA and 5S rRNA graphs represent an average of two measurements. Error bars indicate standard deviation. (B) Quantitative PCR data showing reduced levels of a *tRNAleu* gene in pooled 3-dpf and 5-dpf homozygous *slj* larvae compared with wild-type siblings relative to expression of *hprt*, a Pol II target gene. (C) Reduced levels of the Pol III target gene product 7SL RNA in pooled 4-dpf and 5-dpf *slj* larvae compared with wild-type siblings (as determined by quantitative real-time PCR). *tRNAleu* and *7SL* expression are relative to the expression of the Pol II target gene *hprt*. (D) Northern analyses showing that the levels of mature *tRNAleu* and *tRNAile* transcripts are reduced 48% and 36% relative to *U1 snRNA* (a Pol II target gene) transcript levels in pooled 5-dpf *slj* larvae. This analysis also suggests a small reduction in *slj* 5S rRNA levels, although 5S rRNA levels relative to 5.8S RNA levels were normal when quantified by gel electrophoresis (Figure 5B). (E) Northern analyses showing reduced *pre-tRNAile* levels relative to *U1 snRNA* (unpublished data), in pooled 5-dpf *slj* versus sibling wild-type (WT) larvae. The *pre-tRNAile* transcript is the same size (89 bp) in *slj* and wild-type larvae. (M is a molecular weight marker.) (F) Quantitative real-time PCR data showing reduced *pre-tRNAleu* and *pre-tRNAile* expression relative to the Pol II target gene *hprt* in pooled 5-dpf *slj* versus pooled sibling wild-type larvae.

To determine whether the *slj* mutation disrupted Pol III function, we quantified the levels of Pol III target gene RNAs in wild-type and *slj* larvae. We used an Agilent Bioanalyzer for high-resolution analysis of total RNA (see Materials and Methods). This revealed decreased levels of total tRNAs in 4-dpf and 5-dpf *slj* larvae, but normal 5S and 5.8S rRNA levels (Figure 5A). Normal 5.8S RNA levels in *slj* larvae (unpublished data) were expected because this gene is transcribed by Pol I. Discordant effects of the *slj* mutation on 5S rRNA and tRNA gene expression, which are both transcribed by Pol III, were also not surprising, given similar findings with mutation of yeast Pol III subunit genes [28]. Such differences in the sensitivity of Pol III target genes has been attributed to variable specificity of the Pol III complex for its target gene promoters [28]. Such specificity may also explain the variable effects of the *slj* Pol III defect on tRNA levels (discussed below). However, it is also possible that reduced 5S rRNA transcription and transcription of specific tRNAs in *slj* larvae is compensated by enhanced transcript stability.

To confirm these data, we assessed the effect of the *slj* mutation on individual Pol III target genes. Quantitative RT-PCR experiments showed that there were reduced levels of a tRNA-leu in 3-dpf and 5-dpf *slj* larvae relative to the levels of the Pol II target gene *hprt* (Figure 5B). Interestingly, tRNA-leu levels were normal in 4-dpf *slj* larvae, thus revealing a complex relationship between Pol III activity and target gene transcription. Expression of the 7SL RNA, a component of the signal recognition particle that is also transcribed by Pol III [29], was also reduced in 4-dpf and 5-dpf *slj* larvae (Figure 5C). Reduced levels of mature tRNA-leu and a tRNA-ile were also confirmed by northern analyses of 5-dpf *slj* larvae. Levels of these tRNAs were 48% and 36%, respectively, of their sibling wild-type larvae relative to the expression of the Pol II transcript U1 snRNA on the same blot (Figure 5D). Reduced levels of mature tRNAs suggest reduced efficiency of the *slj* Pol III enzyme, but do not exclude post-transcriptional effects [25,30,31]. Because of the rapid rate at which pre-tRNAs are processed, they are considered to be reliable indicators of Pol III transcription rate [16,32]. Therefore, we used northern blot analysis and quantitative PCR to measure pre-tRNA levels in *slj* and sibling wild-type larvae, relative to the Pol II transcribed genes *U1* snRNA and *hprt* mRNA (Figure 5E and 5F, respectively). The levels of both of the pre-tRNAs examined were reduced in 5-dpf *slj* larvae compared with wild-type siblings, supporting the idea that Pol III transcription is reduced in *slj* mutants.

### Cross-Species Analyses Suggest That the *slj* Mutation Disrupts Interaction of Polr3b (Rpc2) and Polr3k (Rpc11) Subunits

Although high-resolution analysis of the 17 subunit Pol III structure has not been defined, a homology-based model of the nine-subunit core yeast Pol III complex and the structure of an associated subcomplex have been reported [3]. In addition, the 17-subunit complex has been visualized, with some of its subcomplexes localized by electron microscopy [4]. The Pol III structure derived from these studies and the original studies describing the Pol II structure [33,34] suggest a high degree of homology, especially in the largest subunits and their contacts with other subunits (most of which are either shared by Pol II and III, or highly homologous [3,4]). Since Rpc2p is highly homologous to the second largest Pol II subunit, Rpb2p, the Pol II structure provided a guide to how the *slj* deletion might affect Pol III function. Examination of the yeast Pol II structure revealed that the region of subunit Rpb2p corresponding to the Rpc2 *slj* deletion makes contact with Rpb9p (discussed in [4]), a subunit homologous to the Pol III subunit, Rpc11p [24]. This suggested the possibility that the region deleted in *slj* Polr3b might contact the zebrafish Rpc11p ortholog, Polr3k. This idea is supported by two hybrid studies of yeast Pol III that show interaction between Rpc11p and the N-terminal region of Rpc2p [5].

**Figure 6:**
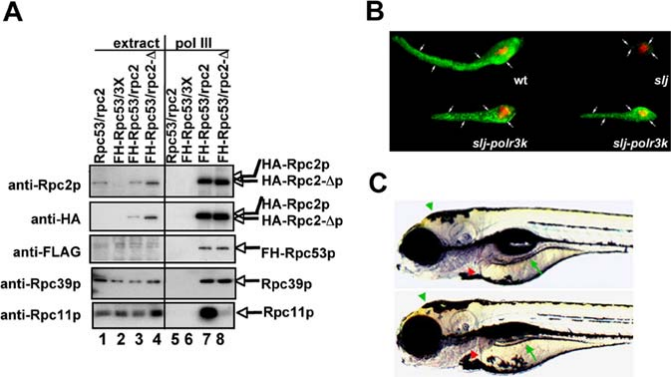
The *slj* Mutation Targets Interaction of Pol III Subunits Rpc2/Polr3b and Rpc11/Polr3k in S. pombe and Zebrafish (A) Deletion of the region between aa267 and aa308 of S. pombe Rpc2 results in a deficiency of Rpc11p in the purified Pol III complex. Wild-type Pol III and mutant Pol III containing *rpc2-Δ*, in which the region between aa267 and aa308 of S. pombe Rpc2 was deleted, were purified from extracts prepared from *FLAG-His6-Rpc53p* strains expressing Rpc2p (“FH-Rpc53/rpc2”), Rpc2-Δp (“FH-Rpc53/rpc2-Δ”), or empty vector (“FH-Rpc53/3X”) as described in Materials and Methods. Mock purification was also performed with control extracts prepared from a wild-type untagged strain (“Rpc53/rpc2”). The input extracts (lanes 1–4) and the purified Pol III (lanes 5–8) were fractionated by 4%–20% sodium dodecyl sulfate (SDS)-polyacrylamide gel electrophoresis and analyzed by immunoblotting using antisera indicated on the left; the detected proteins are indicated on the right. The amounts of input loaded relative to that used for purification are 0.4% lane 1; 0.13% lane 2; 0.1% lane 3, and 0.1% lane 4. A total of 5% of the purified material was loaded in lanes 5–8. (B) Lateral view of the pancreas from a 5-dpf wild-type (wt) larva, a 5-dpf *slj* larva (*slj*), and two 5-dpf *slj* larvae that were microinjected with a *hsp70/polr3k* expression construct (*slj-polr3k*), following heat shock (as described in Materials and Methods), and carboxypeptidase A (green) and insulin (red) immunohistochemistry. Expression of the *polr3k* cDNA from the *hsp70* heat shock promoter induces partial rescue of the slj exocrine pancreas phenotype. (C) Lateral views of a 5-dpf control wild-type (wt) and *polr3k* Morpholino-injected larvae (*polr3k-MO*). The *polr3k* knockdown produces a phenotype that is similar to that caused by the *slj* mutation (Figure 1B). The intestine (green arrow), cranial morphology (green arrowhead), and terminal branchial arches (red arrowhead) are reduced with *polr3k* knockdown.

To further explore whether the *slj* Polr3b deletion might compromise interaction with Polr3k, we engineered S. pombe to express a mutant Rpc2p with a deletion (Rpc2-Δp) corresponding to the evolutionarily conserved region deleted by the *polr3b slj* mutation, and examined its subunit interactions. Hemagglutinin (HA)-tagged versions of wild-type or mutant S. pombe rpc2 were introduced into an S. pombe strain containing a His6-FLAG-tagged Rpc53p subunit, and Pol III complexes containing the tagged subunits were recovered by sequential affinity immunoprecipitation as described in Materials and Methods. Western blot analysis of multiple subunits revealed a markedly reduced amount of Rpc11p in the Pol III complex containing Rpc2-Δp (Figure 6A, lane 8) relative to wild-type Rpc2p (Figure 6A, lane 7). This experiment supports the idea that exon 10 of zebrafish *polr3b* encodes amino acids crucial for the stable interaction of the zebrafish orthologs of Polr3b and Polr3k (Rpc2 and Rpc11), and suggests that the slj phenotype might be due, at least in part, to instability or failure of this interaction in vivo.

### Overexpression of Zebrafish *polr3k* Rescues *slj* Exocrine Pancreas Defects

Pol III subunit interactions in yeast have largely been defined via two-hybrid analyses or the more functional approach, overexpression–suppression experiments. The latter approach involves suppression of hypomorphic phenotypes by overexpression of a gene encoding an interacting subunit. To adapt this approach to the possibility that the *slj* mutation might affect the efficiency of the Polr3b–Polr3k interaction, we injected zebrafish *polr3k* cDNA driven by the heat-inducible *hsp70* promoter, which drives high levels of gene expression throughout the embryo (albeit in a mosaic fashion; [35]) in transient expression assays. This led to partial but highly significant rescue of the *slj* exocrine pancreas defect (Figure 6B; *n* = 8 of 13 and *n* = 9 of 9 injected *slj* larvae rescued from two independent experiments). This rescue, which in most larvae was more pronounced than that achieved with the BAC injection (Figure 2B), is consistent with data on Rpc11p in yeast two-hybrid analyses of Rpc2p-Rpc11p, and the predicted interaction between these subunits in the structures noted above. Suppression of the slj phenotype by microinjection-overexpression of Polr3k provides strong evidence to indicate that the slj phenotype is due, in significant part, to the deficiency of a stable interaction between Polr3k (Rpc11) and Polr3b (Rpc2) in vivo.

Polr3k-mediated rescue of the *slj* exocrine pancreas defects suggests that a decrease in the concentration of Polr3k may produce a phenocopy of the slj phenotype. To explore this hypothesis, we again used antisense Morpholino-mediated knockdown in developing zebrafish, this time targeting Polr3k. Consistent with this idea, injection of a Morpholino targeting the translation initiation codon (5′-ATG) of the zebrafish polr3k mRNA generated an slj phenocopy in approximately 42% of injected embryos (*n* = 31 and 43 embryos analyzed in two independent experiments; Figure 6C). By contrast, control Morpholinos did not generate a slj phenocopy in any of the injected embryos (unpublished data).

## Discussion

The major conclusion that can be drawn from this study is that a mutation in the zebrafish second largest Pol III subunit (Polr3b), that impairs its association with another Pol III subunit (Polr3k), causes tissue-specific defects in zebrafish development. These defects appear to result from failure of adequate proliferation and growth of cells that have a subcritical level of active Pol III. Strikingly, much of the developmental defect in the exocrine pancreas can be overcome in the Polr3b mutant fish by overexpression of Polr3k. These data indicate the importance of the Polr3b–Polr3k interaction in Pol III function, as well as the effect of reduced Pol III activity in tissue-specific development.

Because zebrafish *slj* larvae contain less tRNA and 7SL RNA, as well as other Pol III transcripts, it is presumed that the developmental defects observed in *slj* larvae result from a deficiency of small RNAs that regulate essential cellular processes such as ribosome biogenesis, protein synthesis, and cell-cycle progression [1,6]. This is particularly important to dividing cells, as evidenced by numerous studies showing the effects of reduced translational output on cell growth and cell-cycle progression (discussed in [36]). For example, a 2-fold reduction in tRNAiMet levels is associated with a 3-fold decrease in the doubling time of yeast [37]. Disruption of Pol III transcription can also activate stress-response pathways and indirectly disrupt cross-talk between Pol III and the Pol II transcriptome [38]. Such an effect may also contribute to the developmental defects imparted by the *slj* mutation.

Given the importance of Pol III, it is not surprising that cells contain a number of mechanisms to regulate its activity. Recent studies in mammalian cells have focused on how tumor suppressors, oncogenes, and cell-cycle proteins can either restrict or enhance recruitment of Pol III to its target gene promoters [8–14]. This control may involve up-regulation of the genes encoding Pol III transcription factors, altered protein–protein interactions between the transcription factors, or alternatively, a direct effect on Pol III itself [13,14]. The results reported here are distinguishable from these studies because they suggest that tissue-specific developmental defects in highly proliferative tissues may arise from impaired interaction between two Pol III subunits.

Although deregulated Pol III activity is a common feature of tumorigenic and hypertrophic cell growth in cultured cells [13,14,39], evidence that elevated Pol III activity is required for phenotypic transformation in a multicellular organism has been lacking. Thus, our finding that reduced Pol III transcription in zebrafish *slj* mutants disrupts growth and development that is dependent on cell proliferation in the digestive system, retinae, and other highly proliferative larval tissues is noteworthy. Similarly, whereas there was considerable data showing that reduced tRNA levels appear to limit the proliferation of cultured cells, the present report illustrates the potential importance of this mechanism in a multicellular organism. We found that the Pol III defect imparted by the *slj* mutation had a far more pronounced effect on actively cycling cells than on tissues principally populated by quiescent postmitotic cells, such as skeletal muscle, heart, and the pancreatic islet. These data further support the idea that regulation of Pol III activity is integral to cell growth and proliferation in vertebrate tissues, and moreover, illustrate tissue-specific developmental defects that arise as a result of not meeting the Pol III–dependent demands of such cells. Finally, although it is possible that the development and function of quiescent cell types in *slj* mutants may be sustained by maternally derived Polr3b protein, the metabolic demand of developing cells may exceed maternal supply (as described for genes encoding subunits of the coatomer complex in the developing zebrafish notochord [40]). Thus, the sensitivity of tissue progenitor cells, rather than quiescent cells, to reduced Pol III activity caused by *polr3b* mutation may reflect such metabolic demands.

Although we have not compared Pol III activity levels in the tissues that were differentially affected by the *slj* mutation, our data suggest that the threshold of Pol III activity required to sustain development of different cell types within a specific tissue is variable. We found that intestinal stromal cell proliferation was increased in early *slj* larvae (InS, 48 hpf; Table 1), but was normal at a slightly later stage, when epithelial proliferation is significantly reduced (72 hpf). We also found that the effect of the *slj* mutation varied between different tissues, with a far more pronounced effect on exocrine pancreas development than intestinal development [21–23]. A second possibility that is consistent with our data is that developing cells might require different levels of Pol III activity for their differentiation programs. This is consistent with the finding that although there is normal differentiation of most epithelial lineages in the *slj* intestine, epithelial morphogenesis is abnormal [22].

A third idea to emerge from this work is that vertebrate cells may regulate Pol III activity, in part by regulating its subunit gene expression. Even when the overall expression level of *polr3b* is low, such as on 3 dpf and at later stages, it was consistently most pronounced in the tissues that have a high percentage of proliferative cells, such as the intestine and pancreas (Figure 4). Consistent with this idea, it was previously shown that expression of the gene encoding the Pol III subunit Rpc53/Polr3d (also known as *BN51*) increases in response to serum stimulation of cell proliferation [41].

### Conserved Pol III Subunit Interactions in Yeast and Zebrafish

Mutants targeting yeast Rpc2p had been isolated by a genetic screen that selected for impaired termination by Pol III, and were later found to affect elongation rate with reciprocal effects on termination [42–45]. Because the *slj* mutation was adjacent to some of these yeast *rpc2* mutations, and because *rpc11* was linked to Pol III termination and RNA 3′-end formation [24,25], we wanted to know whether termination was affected in *slj* mutants. A minimal efficiency vertebrate Pol III terminator consists of a run of four thymidine (T) residues, with termination efficiency increasing as the number of T residues increases [46]. Transcription beyond a weak Pol III terminator in vivo can be visualized on northern blots as pre-tRNA transcripts that extend beyond the 3′ terminator into flanking DNA [47]. To examine this possibility, we identified a *tRNAile* gene with a minimal 4T terminator and examined its transcripts in wild-type and *slj* mutant fish. Using an intron probe as well as a 3′ flanking probe designed to detect read-through transcripts that extend beyond the terminator, we found no evidence of a difference in the Pol III termination efficiency of this gene in wild-type and *slj* larvae, since the pre-tRNA-Ile transcript size was identical in *slj* and wild-type larvae, with no evidence of longer transcripts on the blot (Figure 5E and unpublished data). These data are consistent with the finding that function-altering mutations in *rpc11* did not affect termination efficiency in fission yeast [25] and a revised role for Rpc11p in Pol III recycling rather than termination per se [26]. Recycling, or facilitated reinitiation, is a feature of the high efficiency of Pol III [26]. Given evidence of no impairment of termination by *slj* Pol III, we speculate that reduced reinitiation may be the cause of decreased Pol III transcription in *slj* mutants.

Contrary to what might be expected from the study of a *Saccharomyces cerevisiae rpc11* mutant that contained wild-type S. pombe Rpc2p [24], Pol III purification from S. pombe revealed similar Rpc53p levels in the wild-type and *slj* Rpc2-Δp cells (Figure 6A). This may be explained by the fact that the genetic approaches differed (small deletion in Rpc2 in S. pombe, versus replacement of S. cerevisiae Rpc11 with S. pombe Rpc11 in S. cerevisiae) and that very different Pol III purification schemes were used (epitope tag affinity chromatography versus extensive ion exchange chromatography) in the present and prior studies, respectively.

In summary, the work described in this study reveals an unexpected importance of the Rpc2–Rpc11 interaction during development and demonstrates the utility of the zebrafish system. We have shown that in vivo analyses of Pol III function are feasible in zebrafish and that they complement analyses in other model systems. We have also shown that the zebrafish may be used to reveal the effects of disrupting Pol III in complex multicellular tissues. An idea suggested by these studies is that specific cell types within a multicellular organism may require different levels of Pol III activity and that this may reflect their rate of proliferation and/or growth. Thus, it is conceivable that selective Pol III inhibitors may be able to target metabolically active cells in proliferative or hypertrophic disease states.

## Materials and Methods

### Positional cloning of *slj* locus.

The *slj* mutation bulk-segregant analyses identified two Chromosome 18 markers linked to the *slj* locus (M. Mohideen, M. Fishman, and M. Pack; unpublished data). Subsequent analyses identified two closely linked markers, z15417 and z21330, that were 0.08 cM and 0.02 cM from the *slj* mutation (three recombinants out of 3,612 meioses and one recombinant out of 4,186 meioses). A BAC clone zK130I16 spanning the critical region bounded by these markers was identified. Within this BAC, two additional polymorphic markers, CA11 and GT14, were identified. Meiotic mapping showed that one and zero of 1,806 *slj* larvae were recombinant for these markers. Sequence analyses indicated that the two markers, CA11 and GT14, were located within intron 23 and intron 10 of the *polr3b* gene, respectively.

### BAC rescue experiments.

To verify that BAC clone zk103i16 spanned the *slj* locus, phenotype rescue experiments were performed. BAC DNA was prepared using a commercially available kit (PSI Clone BAC DNA kit; Princeton Separations). The BAC DNA (1 nl of 12.5 ng/μl in 0.1% phenol red) was microinjected into the progeny of a *slj*/+ intercross at the one-cell embryonic stage. Equivalent numbers of embryos were injected with phenol red solution as control. The embryos were raised to 4 dpf and assayed by anti-carboxypeptidase A and anti-insulin immunohistochemistry as described [21–23].

### Morpholino injection.

Morpholinos targeting the *polr3b* and *polr3k* mRNAs were injected into newly fertilized one-cell to four-cell stage zebrafish embryos as previously described [48]. The sequences of the Morpholinos are:

*polr3k* ATG: CAGGAGCATTTTCAAACAGTCATAG

*polr3k* control: TTCAAGTTTCATTTGTTTACCTGCA

*polr3b* ATG: TTTCCCCGAACTCCTCTTGCAGCAT

*polr3b* splice: TCCACTCCCATAGCCTGACGAAAGA

### *polr3k* rescue experiments.

For the *polr3k* expression construct, PCR primers were designed that were complementary to the 5′ and 3′ regions of the zebrafish *polr3k* ortholog identified in the zebrafish database. The forward and reverse primer sequences are GATATCGTTTGAAAATGCTCCTGTTTTG and ACTAGTAAGTGAATGATCTGGTTATGC.

The primers were used to amplify cDNA derived from wild-type 5-dpf zebrafish larvae. The predicted Polr3k protein consists of 108 amino acids, of which 102 are either identical or similar to the mouse Polr3k protein. The cDNA was cloned downstream of the zebrafish *hsp70* heat shock promoter [48]. The construct was microinjected into the progeny of heterozygous *slj*/+ matings at the one-cell stage. Heat shock was performed approximately every 8 h beginning at 48 hpf through 84 hpf. Heat-shocked and control larvae were processed for carboxypeptidase A immunohistochemistry as described [22].

### Genomic organization of the *polr3b* locus.

To assemble the zebrafish *polr3b* cDNA sequence containing the longest open reading frame, a comparative analysis of the predicted amino acid sequences of zebrafish, human, and mouse Polr3b proteins was performed. This showed that zebrafish Polr3b protein had 98% and 96% similarity to the human and mouse Polr3b proteins, respectively (unpublished data). Primers (RPC2-1FA and RPC2-2RB) flanking the coding region of zRPC2 were designed from the conserved cDNA sequences and used for RT-PCR with total RNA derived from 5-dpf zebrafish larvae. A 3,514-bp fragment was amplified and sequenced. The comparison of the cDNA sequence of zebrafish *polr3b* to the corresponding genomic sequence indicated that this gene consists of 28 exons and 27 introns spanning a 38.5-kb genomic sequence. The longest open reading frame is 3,393 bp, and the predicted protein consists of 1,131 amino acids with a molecular weight of 127.8 kDa.

Sequence analysis of the *polr3b* cDNA from separate pools of homozygous wild-type and *slj* larvae revealed complete or partial deletion (121 and 63 bp, respectively) of sequence derived from exon 10 encoded by the *slj* and not the wild-type *polr3b* allele. Sequence analyses of exon 10 splice donor and intron 10 splice acceptor revealed a thymidine-to-cytosine transition of genomic fragments amplified from *slj* larvae, but not homozygous wild-type sibling larvae.

### RNA in situ hybridization, immunohistochemistry, and histology.

BrdU and PH3 immunohistochemistry were performed as previously described [21,22]. Cell counts were performed using histological sections of whole-mount specimens. For BrdU counts, the number of intestinal epithelial or stromal cells analyzed per embryo ranged between 350 and 775. For the exocrine pancreas, 50 cells per embryo were counted. Three wild-type and three *slj* larvae were analyzed for each time point. Histology and whole-mount RNA in situ hybridizations were performed as previously described [21,22].

### Quantitative real-time PCR.

Quantitative real-time PCR (RT-PCR) was performed as previously described [49]. RT-PCR was performed using total RNA derived from 30 or more pooled 2–5-dpf wild-type and sibling *slj* larvae that were identified either morphologically or molecularly. For measurements of total tRNA, 5S rRNA, and 5.8S rRNA levels, total RNA was separated electrophoretically using an Agilent Bioanalyzer. The chromatographic image was analyzed with image analysis software (NIH Image 3) for transcript quantification. For northern analyses, total RNA from pooled wild-type and sibling *slj* larvae were used using standard techniques. Primers used in quantitative PCR experiments are:

*tRNAleu*: GTAGGATGAACTGAGTTTTAA; AAAGGCAGAAGAGAACTGGTTTATT

*7SL RNA*: TTCGGTATCGATATGGTGCTC;  GCTTTGACCTGCTCCGTCT

*pre-tRNAleu*: AGAATGGCCGAGTGGTCTAA; CCAGCTGGAGACCAGAAATC

*pre-tRNAile*: CGCGCGGTACTTATAAGACAAT; GAACTCACAACCTCGGCATT.

### Engineering of S. pombe rpc2 mutant.

The S. pombe FLAG-tagged version of rpc2^+^ was cloned by PCR using genomic DNA and two primers (SPREPFOR1: 5′-GCTAGTCGACATGGATTACAAAGACGATGACGACAAGGGGGTAAATACTGC;5′-GCTAGTCGACATGGATTACAAAGACGATGACGACAAGGGGGTAAATACTGC; and SPREPREV: 5′-ATACCCGGGTCAATACTTAAATTCGT). The PCR products were digested with SalI and SmaI, and cloned between the XhoI and SmaI sites of pREP3X, yielding pREP3X-rpc2. The region spanning amino acid (aa)267 and aa308 of Rpc2p, which corresponds to aa240 and aa380 of the zebrafish Rpc2 ortholog, Polr3b, which was deleted in the *slj* mutant, was deleted by the QuikChange XL Site-Directed Mutagenesis (Stratagene) using pREP3X-rpc2 and two primers (RPC2F: 5′-CAGTGTAGCAGATGATATTCCTATAGTGGTTGTTTTAAAAGCATTAGAATATATCGGTGCGCGTGTTAAGG; and RPC2R: 5′-CCTTAACACGCGCACCGATATATTCTAATGCTTTTAAAACAACCACTATAGGAATATCATCTGCTACACTG). The resulting *Rpc2* mutant was called *Rpc2-Δp*. To replace a FLAG tag with an HA tag in Rpc2p and Rpc2-Δp, PCR was performed with primers retremfor3 (5′-ACGGTCGACATGTAC CCATACGACGTTCCAGACTACGCTGGGGTAAATACTGCC GGA) and SPREPREV. The PCR products were cut with SalI and SmaI, and cloned between the XhoI and SmaI sites of pREP3X, producing pREP3X-HA-rpc2 and pREP3X-HA-rpc2-Δp, respectively. To purify wild-type Pol III and Pol III mutants containing Rpc2-Δp, pREP3X-HA-rpc2, and pREP3X-HA-rpc2-Δp were transformed into yYH3282(h+, his3-D1, leu1–32, ura4-D18, ade6-M216 ret1D::[FH-rpc53, ura4+]), in which rpc53 is tagged with FLAG and six histidine residues. Purification of wild-type and mutant Pol III was carried out as previously described [50] with the following modifications: the eluate from the Ni-NTA column was incubated with 20 μl of anti-HA beads for 4 h at 4 °C; the bound proteins were eluted by boiling the beads in 2× tris-glycine SDS gel loading buffer.

## Supporting Information

### Accession Numbers

The GenBank (http://www.ncbi.nlm.nih.gov/Genbank) accession numbers for the proteins discussed in the paper are as follows: human Polr3b (NM_018082) and mouse Polr3b (NM_027423).

## Abbreviations

aa: amino acid
BAC: bacterial artificial chromosome
BrdU: bromodeoxyuridine
dpf: days post-fertilization
hpf: hours post-fertilization
PH3: phospho-Histone H3
Pol III: RNA polymerase III
rRNA: ribosomal RNA
RT-PCR: real-time PCR
*slj*: *slim jim*
snRNA: small nuclear RNA
tRNA: transfer RNA
